# Protracted amygdalar response predicts efficacy of a computer-based intervention targeting attentional patterns in transdiagnostic clinical anxiety

**DOI:** 10.1038/s41398-019-0458-x

**Published:** 2019-03-28

**Authors:** Mary L. Woody, Jamie O. Yang, Logan Cummings, Danielle Gilchrist, Simona Graur, Greg J. Siegle, Rebecca B. Price

**Affiliations:** 1 Department of Psychiatry, University of Pittsburgh, Pittsburgh, PA USA; 2 David Geffen School of Medicine at University of California, Los Angeles, CA USA; 3 Florida International University, Miami, FL USA; 4 University of Pittsburgh Medical Center, Pittsburgh, PA USA

## Abstract

Individuals with clinical anxiety demonstrate an attention bias toward threatening information, which is thought to be partially driven by heightened amygdala activity to perceived threat. Attention Bias Modification (ABM) is a computer-based treatment that trains attention toward neutral stimuli and away from threatening stimuli. Alterations in initial processing of threat have been linked to ABM responses, but the impact of protracted processing in the aftermath of neutral and threatening information on ABM outcomes has not been well studied. Our study tested whether sustained activity in the amygdala, which occurred after neutral and threatening stimuli had been removed, could predict which individuals would respond well to ABM. Unmedicated anxious individuals underwent a baseline fMRI assessment during performance of a task sensitive to protracted emotional processing. Afterward, they were randomized to complete eight sessions of ABM (*n* = 38) or a sham training (*n* *=* 19). ABM patients who displayed greater sustained bilateral amygdalar response in the aftermath of neutral stimuli displayed the least improvement in self-reported (but not clinician-rated) vigilance symptoms. In contrast, amygdalar response did not predict improvement in sham patients. Results suggest that in certain anxious individuals, the amygdala may have a robust protracted response even to subjectively neutral cues, which could make these individuals a poor fit for ABM because of its focus on repeatedly retraining attention toward neutral cues. Findings may help elucidate neural mechanisms of ABM and promote the identification of a subset of anxious patients who would be good candidates for this intervention.

## Introduction

Clinical and subclinical forms of anxiety represent a significant public health burden^1,2^, but response rates for current first-line treatments for anxiety stand at only 50–70% with high rates of relapse and low rates of remission^3–6^. Some of the most deleterious transdiagnostic symptoms of anxiety involve involuntary orientation toward and perseveration about potential threats (i.e., vigilance)^7^. Thus, the development of novel mechanistic approaches to reduce vigilance may be an essential next step in advancing the treatment of anxiety disorders.

Among anxious individuals (as a transdiagnostic group), vigilance often manifests at the behavioral level in the form of an attentional preference for threatening information, or an attentional bias (AB)^8^. Specifically, anxious individuals orient attention more quickly toward threatening stimuli than neutral stimuli, indicating an AB toward threat^9^. This observation invited speculation that AB may be a mechanism underlying the development and maintenance of anxiety disorders. Therefore, Attention Bias Modification (ABM) treatment was developed to therapeutically exploit the potentially causal role of AB upon anxiety. ABM treatment seeks to modify AB by training patients to preferentially attend to non-threatening stimuli, rather than threatening stimuli, in the initial stages of attention orientation. In a seminal study, MacLeod and colleagues found that ABM was successful in modifying AB and that this AB modification reduced stress reactivity^10^. Following this study, a continually growing literature suggests that ABM treatment is effective in reducing anxiety symptoms^11–13^. However, subsequent meta-analyses examining the effectiveness of ABM treatment show that its potential beneficial effects on anxiety are inconsistent across individuals and studies^14^. In addition, anxious individuals as a group show a significant but small AB suggesting that there is clinically-relevant variability in AB across anxious individuals^9^. Thus, more research is necessary to understand *which* anxious patients will most likely benefit from ABM and *why* they do, since anxiety disorders affect a heterogeneous group of individuals.

To date, ABM studies have largely focused on group-level observations of whether anxious patients, as a group, benefit from ABM, which can mask considerable within-group heterogeneity linked with ABM treatment outcome. To understand which anxious patients are most likely to benefit from ABM, one important consideration may be the timeframe over which a given anxious individual exhibits AB toward threat. As a group, anxious individuals exhibit vigilance to threat during initial stages of processing (e.g., 16–500 ms after stimulus onset)^9^. Thus, an individual differences approach that examines the timing of threat processing may capture critical, clinically relevant information. For example, prior research has shown that patients who exhibit larger baseline transient neural responses to threatening stimuli across a range of cognitive-affective brain regions display the greatest reductions in clinician-rated vigilance following ABM treatment^15^. This work suggests that patients who display an initial preferential response toward threat in AB-related brain regions are best suited for ABM treatment, which is consistent with prior behavioral work that has shown that initial processing of threat is linked to ABM treatment responses^13,14,16^. However, some anxious individuals also exhibit protracted threat processing even after the stimulus has been removed^17^. Although it is yet to be established empirically, protracted processing in the aftermath of neutral and threatening information could also potentially impact outcomes following ABM treatment given that ABM is only designed to target AB during early stages of attention and while the threat stimulus is still present on the screen. Matching the timescale of intervention mechanisms is critical because if there is a mismatch between the time frame of AB that is shown by a given individual and the time frame of AB that is targeted with ABM, then the result will likely be a worse clinical outcome.

Research examining other internalizing disorders, such as depression, has shown that protracted neural processing in the aftermath of emotional stimuli can be an effective predictor of treatment response^18^. For example, Siegle and colleagues found that patients who exhibited greater sustained amygdalar responses in the aftermath of negative stimuli displayed greater improvement following cognitive-behavioral therapy^19^. Because amygdalar response to emotionally salient stimuli is thought to be a transdiagnostic marker of internalizing disorders^20,21^ and prominent clinical phenotypes of anxiety involve protracted forms of attention (e.g., worry)^22,23^, it is possible that sustained amygdalar response in the aftermath of emotional stimuli could also be utilized to predict the effectiveness of interventions for anxiety, such as ABM.

Building from the identified gaps in previous research, the current study asked specifically whether protracted activity in the amygdala in the aftermath of neutral and threatening stimuli could predict who would be a good candidate for ABM. In addition to its potential effectiveness in predicting treatment response^18,19^, the amygdala was chosen as it is a well-replicated, key player in the neural circuitry implicated in threat processing in anxiety disorders^24^. Specifically, the amygdala acts as the central fear processor of the brain and initiates responses that encode the salience and affective properties of the stimuli and promote orientation toward fear-related stimuli^25^ as well as playing a critical role in protracted, ruminative attention to negative information^26,27^. Among anxious individuals, increased activation of the amygdala is broadly implicated in attentional bias towards threat^28,29^. Notably, in the context of anxiety, altered patterns of amygdalar response over time are not restricted to negative stimuli alone. Of particular interest here, anxious individuals sometimes exhibit protracted amygdalar responses to both neutral and threatening stimuli, which is thought to reflect an overgeneralized perception of potential threat^30,31^. Although it is yet to be tested, taken together, these studies strongly suggest that protracted amygdalar response to negative and/or neutral stimuli could be a successful predictor of ABM treatment outcomes.

The primary aim of the current study was to examine protracted amygdalar responses to both threatening and neutral stimuli as a neural predictor of success in ABM treatment. Of note, this study is one of the first to examine neural predictors of ABM response and is the first of which we are aware to extend the literature to examine amygdalar responses during a protracted emotion processing task. Because prior work has suggested that the amygdala plays a central and specific role in protracted processing of threat, we exclusively examined amygdalar responses. Critically, this decision reduced the need for multiple comparisons correction across the whole brain, which presents challenges for individual differences analyses of slow event-related timeseries data (i.e., where each timepoint requires its own Type 1 error correction). Together, these factors generated compelling a priori hypotheses specific to the amygdala. To measure protracted amygdalar responses, we utilized a protracted emotion processing task that has been well-validated in the context of depression research^19,26,32^.

Patients across multiple diagnostic categories were recruited for the current study, as transdiagnostic approaches have become increasingly important in advancing the field of psychiatry by better representing the real-world clinical patient population^33^. Using a randomized controlled design, patients with transdiagnostic clinical anxiety were assigned to either active ABM or a sham training. Before the onset of treatment, patients completed the protracted emotion processing task, which quantified amygdalar responses in the aftermath of both neutral and threatening idiographically chosen stimuli in an fMRI scanner with the explicit goal of identifying which subset of anxious patients would be good candidates for ABM. Because ABM is only designed to reduce AB to threat stimuli at transient (rather than protracted) stages and only when threat stimuli are currently presented on the screen, we predicted that patients who exhibited greater protracted amygdalar responses in the aftermath of threat words would be a relatively poor fit for the mechanistic target of ABM and thus would display the least improvement with ABM (as defined by both clinician-rated and self-reported symptoms of vigilance). Similarly, because ABM retrains attention toward neutral stimuli, we hypothesized that patients who exhibited greater protracted amygdalar responses in the aftermath of neutral words would also be a poor mechanistic fit and display the least improvement with ABM.

## Materials and methods

### Participants

Patients were 70 unmedicated adults reporting clinical levels of transdiagnostic anxiety and associated clinician-rated disability (full inclusion/exclusion criteria in Supplement). See Table 1 for demographic and clinical characteristics. Patients were randomized to receive ABM (*n* = 49) or a sham training (*n* = 21) (clinicaltrials.gov: NCT02303691). Uneven allocation to the ABM versus sham conditions was used to allocate a greater proportion of available funds towards characterizing the ABM sample in an effort to enhance statistical power in the active ABM group, as the primary study aims focused on mechanistic predictors of ABM response. The sham condition was included so that we could probe the specificity of our results to active ABM, though power was constrained to detect effects statistically moderated by condition. 94% of randomized patients completed their assigned treatment condition and the post-treatment assessment (CONSORT diagram: Supplement). This study was approved by the local Institutional Review Board and informed consent was obtained from all patients. Please note, this sample has also been used in analyses from a prior published study^15^.

**Table 1:** Demographic and Clinical Characteristics of the Sample

	ABM (*n* *=* 39)		Sham (*n* *=* 19)	
	Pre-treatment	Post-treatment	Pre-treatment	Post-treatment
Demographics:				
Caucasian, *n* (%)	24 (62%)	–	14 (74%)	–
Female, *n* (%)	29 (74%)	–	14 (74%)	–
Age	29.72 (8.48)	–	30.74 (12.13)	–
Primary outcome measures				
MASQ: Anxious Arousal	32.97 (10.99)	28.54 (9.75)	33.47 (11.04)	29.90 (13.61)
CAPS: Vigilance	4.72 (2.01)	4.08 (1.98)	5.21 (2.30)	4.05 (2.07)

Data presented as mean (SD) unless otherwise noted. *MASQ* Mood and Anxiety Symptoms Questionnaire, *CAPS* Clinician-Administered PTSD Scale

### Measures

#### ABM and Sham conditions

Patients and clinical assessors were blind to treatment assignment. The ABM and sham conditions were modeled after prior studies^11^ and described in detail by our group in a previous publication^15^. Briefly, patients in both conditions completed eight twice-weekly laboratory-based sessions, which used a modified dot-probe task to retrain attention. At the baseline assessment, ten idiographic threat words that captured the primary foci of anxiety were selected collaboratively by the patient and clinical interviewer. These idiographic threat words were matched for familiarity and word length to 10 neutral words from a normative corpus used previously in ABM research^11^. Twenty additional general threat words and 20 additional neutral words from the same normative corpus were used as training stimuli in an effort to promote broadly generalized attentional retraining. During training trials (300 administered at each training session), word pairs (80% threat-neutral; 20% neutral-neutral) were presented vertically for 500 ms, followed by a probe (“E” or “F”) in either the upper or lower word location. Patients responded via button press to indicate the probe letter displayed.

The ABM condition was identical to the sham condition except for the relation between the probe location and the threat word in the threat-neutral trials. In ABM, for 100% of threat-neutral trials (80% of all trials), the probe replaced the neutral word in a threat-neutral pair, thereby shaping attention away from threatening cues through practice. In the sham condition, the probe replaced either the threat or neutral word with equal likelihood.

#### Clinical outcome measures

Outcome measures were collected at two timepoints: at a pre-training baseline visit (completed approximately 1–2 weeks prior to the beginning of attention training) and at a post-training visit (within approximately 1 week of completion of the final computer training session). Residual symptom scores post-treatment (regressing out pre-treatment scores) were calculated within each treatment group, where lower numbers indicate a greater decrease in symptoms (more favorable outcome) relative to other individuals in the same treatment group. Conversely, a higher score indicates symptoms remained high after treatment relative to other individuals in the sample. To assess primary clinical outcomes, the study employed both self-report and clinician-rated outcome measures of vigilance. See Supplement for additional information and analyses regarding secondary clinical outcomes.

The primary self-report outcome for the trial was the Anxious Arousal subscale from the Mood and Anxiety Symptoms Questionnaire (MASQ; 64-item short form). The MASQ is a well-validated questionnaire that assesses the severity of anxious symptoms and allows discrimination between anxiety symptoms and general distress, with the latter being common across a range of internalizing and externalizing disorders^34,35^. The Anxious Arousal subscale was of principle interest given its capacity to capture clinically relevant symptom patterns of anxious vigilance within transdiagnostic disorders. In the current study, internal consistency for the MASQ anxious arousal subscale was good (*α* = .88).

The primary clinician-rated outcome for the trial was the “hypervigilance” item of the well-validated Clinician-Administered PTSD Scale (CAPS-vigilance)^36^, which sums two sub-items assessing frequency and intensity of vigilance (e.g., “have you been especially alert or watchful” for threat-related information or “felt as if you were constantly on guard?”). To ensure transdiagnostic relevance of this measure, assessors were trained to provide idiographic examples of vigilance that were relevant to the participant’s principle anxiety domains (e.g., were you “on guard” for signs of negative social evaluation, health/monetary/safety concerns, interoceptive panic cues, etc.). To assess inter-rater reliability, a random subset of videotaped interviews (15%) was scored by a second rater, and 100% reliability was obtained.

#### fMRI task and data acquisition

##### fMRI acquisition

T2*-weighted images depicting BOLD contrast (TR = 2000 ms; TE = 27 ms; flip angle = 80°; 38 slices; 3.125 × 3.125 × 3.2 mm voxels) were acquired on a 3 T Siemens Trio. Visual stimuli were presented on a rear projection screen connected to a computer running E-Prime and viewed through a mirror attached to a head coil. The patient responded to stimuli using a 5-button glove connected to the computer. Standard preprocessing steps were applied using Analysis of Functional Neuroimaging (AFNI) including slice time correction, motion correction, linear detrending to correct drift, outlier rescaling, temporal smoothing, spatial smoothing, and nonlinear warping to the Montreal Neurological Institute Colin-27 brain set. Patients with excessive motion during the task (>30% of scans showed incremental movement > 1 mm or incremental rotation > 1°, or >30% of scans showed absolute movement from baseline > 5 mm or absolute rotation > 5°) were excluded from analysis (*n* = 2). Data on this fMRI task were not acquired from an additional five subjects due to scanner time constraints. Critically, excluded subjects (*n* *=* 7) did not differ significantly from included subjects on any study variable.

##### Protracted emotion processing task

**Fig. 1:**
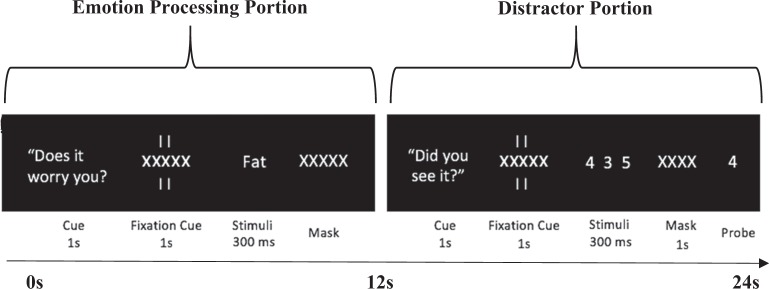
The experimental protocol showing the time-course for the Protracted Emotion Processing Task. Each trial was 24 s, with the emotion processing and distractor portion lasting 12 s each

The Protracted Emotion Processing Task was adapted from Siegle and colleagues^26,32^. As depicted in Fig. 1, the task starts with the emotion processing portion of the task, which consists of the prompt “Does it worry you?” displayed for 1 s; followed by a fixation cue (a row of X’s flanked by vertical lines) for 1 s, followed by presentation of a neutral or threat word for 300 ms, followed by a backward mask (row of X’s) for the remainder of the 12 s period. Both threat (*n* = 15) and neutral (*n* = 15) trials were presented, using words drawn from the patient’s idiographic threat and neutral word lists, respectively (a random 50% of the words from each list were repeated a second time after each list had been presented in full) (see Supplement for details of how idiographic words were chosen). Patients were instructed to rate the worry level of the presented word using buttons assigned for “Yes,” “Somewhat,” and “No.” A reminder of button order (“Y-S-N” or “N-S-Y”, counterbalanced across subjects) was presented in the top right corner of the screen throughout the entirety of those trials. Additional details on how subjective behavioral worry ratings were calculated and analyzed can be found in the Supplement. On every trial, the emotion processing portion of the trial was then immediately followed by a non-emotional distractor portion where patients completed a digit memory task. By drawing the patient’s attention toward a non-emotional memory task during the second half of each trial, the task was optimized to capture protracted neural processing that persisted in the aftermath of the worry prompt, even after competing cognitive information was introduced. The digit memory task contained a cue “Did you see it?” (1 s), a fixation cue (1 s), a series of 3 digits displayed for 1 s each in quick succession, a backward mask (1 s), and a probe digit (target or nontarget) that remained onscreen for the remainder of the 12 s task. Patients were told that when the series of 3 digits appeared, they should try to remember each digit in the series. When the probe digit appeared, they should push buttons for “Yes” or “No” to indicate whether the probe digit was present in the target set.

#### Analytic plan

**Fig. 2:**
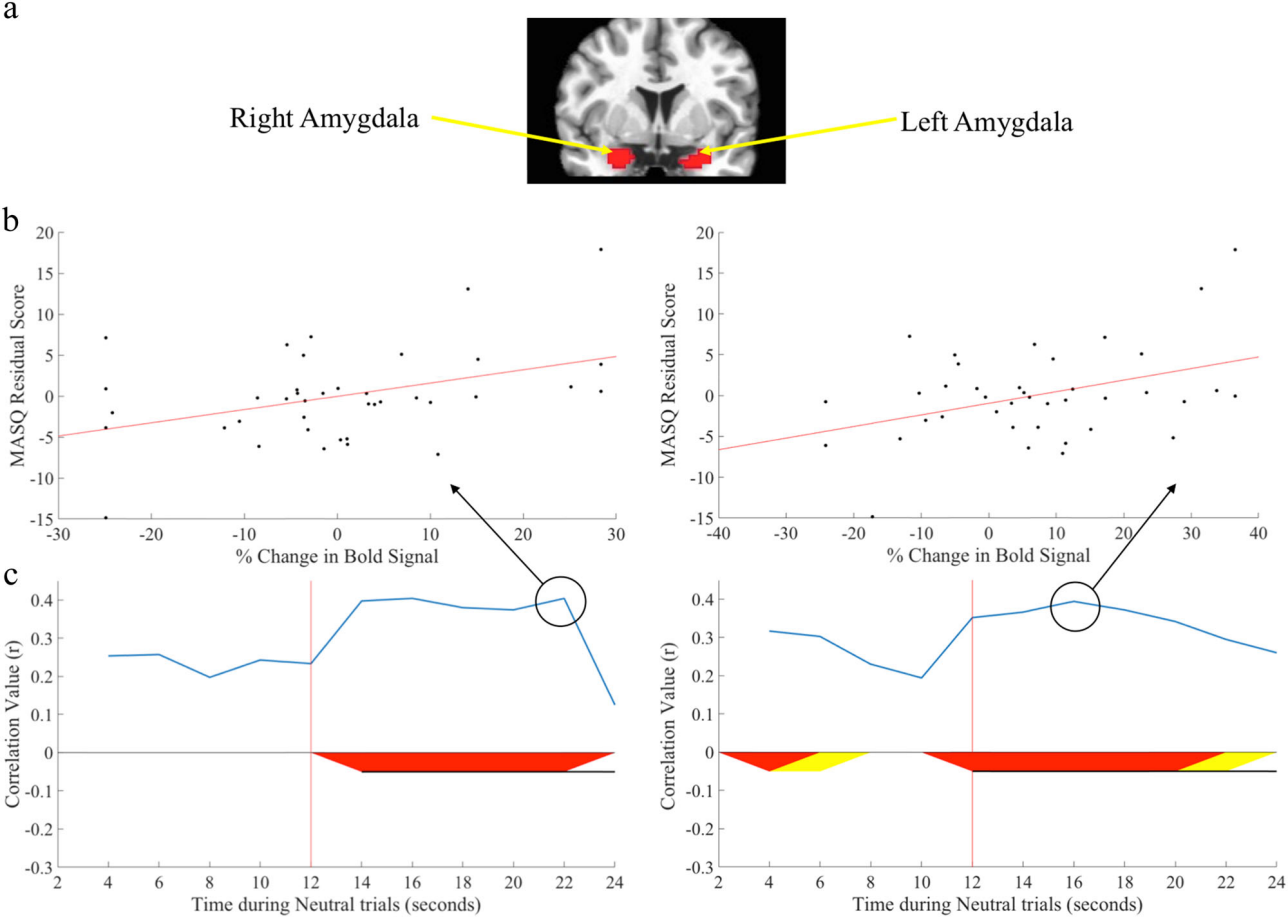
**a** Coronal view of the right and left amygdala ROIs. **b** Scatter plots of the highest correlation coefficient between % change in BOLD activity in the right and left amygdala during neutral trials with MASQ Anxious Arousal residual scores. **c** Correlation coefficient (*r*) value between MASQ Anxious Arousal residual scores and BOLD activity in the left and right amygdala across the full time course of neutral word trials. The vertical red line at 12 s separates the emotion processing portion of the Protracted Emotion Processing Task from the digit memory portion. Time points at which correlation coefficients were considered significant are highlighted in red and yellow on the x axis. Yellow indicates a correlation value of *R* > 0.27 corresponding to a significance level of *p* < 0.10, while red indicates a stronger correlation value of *R* > 0.32 corresponding to a significance level of *p* < 0.05. Correlations were considered robust if they persisted at *p* < .05 for at least four consecutive time points and were indicated by a horizontal black line underneath the red or yellow area

We chose two a priori brain regions of interests (ROIs), the left and right amygdala, known to be involved in anxiety and processing of threat. These two ROIs were defined using the Automated Anatomical Labeling atlas (AAL)^37^. The coronal view of these two ROIs is displayed in Fig. 2a. The 24 s Protracted Emotion Processing Task trials contained a total of 12 time points. BOLD responses for each amygdala ROI were averaged across all task trials of a given emotional type (neutral and threat) at each of the 12 time points (see Fig. 2), and across all voxels within each ROI. To assess whether activity in these ROIs could predict the degree of improvement in post-ABM symptoms, we examined the correlation between BOLD responses at each time point across the time series and each patient’s residual symptom scores. Correlation coefficients were plotted over time, and significant correlations (*r*s *>* .32; *p* < .05) were considered robust if they persisted for at least four consecutive time points. This time-region was identified using Guthrie and Buchwald’s technique to control for Type 1 error in timeseries data^38^.

## Results

Of the 70 patients who qualified for the study, 94.29% (*n* = 66) completed their assigned treatment condition and returned for post-treatment assessment. Of these, 59 patients (*n* = 40 in the ABM condition; *n* *=* 19 in the sham training condition) had usable fMRI data within motion limits. One patient in the ABM condition was excluded due to the onset of psychosis during the treatment phase, leaving 58 patients for analyses. Prior published work from this sample showed pre-to-post treatment symptom decreases across a range of clinician-rated and self-report variables, with effect sizes generally favoring ABM over sham (15; additional details in the Supplement).

### Split-half reliability of amygdalar responses

The split-half reliabilities of left and right amygdalar responses during neutral and threat trials (averaged across time points) were good to excellent (Spearman-Brown coefficients ranged from 0.83 to 0.90; see also Supplemental Table S1).

### Associations of Amygdalar responses with primary clinical outcomes

#### MASQ anxious arousal

**Fig. 3:**
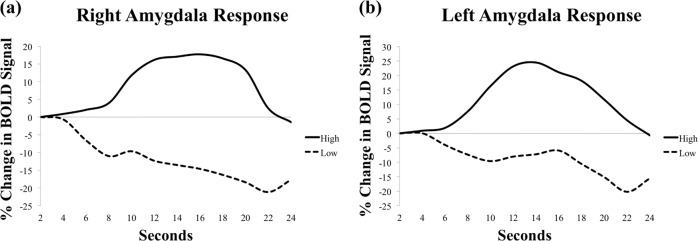
To visually depict the temporal pattern of activity in the right (**a**) and left (**b**) amygdala between individuals displaying high versus low amygdalar response to neutral words, we used a median split of average amygdalar response during time periods that were significantly related to MASQ Anxious Arousal residual scores to divide “high” versus “low” amygdala responders. We then plotted each group’s average % change in BOLD activity over time to visualize the time course of amygdalar responses to neutral words among individuals with high versus low amygdalar response to neutral words

Activity in both the left and right amygdala during *neutral* trials was significantly associated with reduced anxious arousal following ABM. Specifically, left amygdala activity following the onset of the distractor portion of the task (i.e., from 12–20 s into the trial) was significantly and positively associated with MASQ anxious arousal residual scores. Similarly, right amygdala activity following the onset of the digit memory task (i.e., from 14–22 s into the trial) was significantly and positively associated with MASQ anxious arousal residual scores. These findings are depicted in Fig. 2b, c. Figure 3 provides a visual depiction of how activity in the right and left amygdala progressed over time among those exhibiting high versus low reactivity to neutral words. In contrast, neither left nor right amygdala activity during *threat* trials was significantly associated with reduced anxious arousal following ABM. Finally, to explore specificity of findings to the ABM group, we repeated these analyses in the sham condition. We used lenient significance test thresholding to offset the reduced power due to smaller sample size in the sham condition (i.e., correlations coefficients were marked as significant if *r*s *>* .32 for at least four consecutive time points, consistent with significance testing in the ABM condition). For both the left and right amygdala, there were no significant correlations between amygdala activity and anxious arousal MASQ residual scores across sham patients during the neutral or threat trials. These findings are depicted in Figure S1 of the Supplement.

#### CAPS

Neither left nor right amygdala activity during neutral or threat trials was significantly associated with reduced scores on the CAPS (consecutive *r*s *<*.32).

### Exploratory analyses

Because it is possible that amygdalar responses to neutral and threat words during the Protracted Emotion Processing Task was merely a concomitant of self-reported worry about the words, we conducted exploratory analyses to determine if patients’ amygdala activity during neutral and threat trials was related to how much the idiographic neutral and threat words worried them. Neither left nor right amygdala activity during neutral or threat trials was significantly associated with self-reported worry about neutral or threat words, respectively (consecutive *r*s *<* .32). In addition, ratings regarding levels of worry about neutral and threat words were not significantly correlated with either primary outcome measure (please see Supplement for details).

Finally, given prior work from this sample that suggested that transient amygdalar response to threat stimuli significantly predicted ABM response^15^, we conducted exploratory analyses to determine if the significant predictors of ABM response in the previous study versus the current study were distinct. We found that the significant amygdalar predictors of ABM response from^15^ were not significantly correlated with the significant amygdalar predictors from the current study (lowest *p* *=* .317).

## Discussion

The primary aim of the current study was to determine whether individual differences in protracted amygdalar response in the aftermath of threatening and neutral stimuli could predict ABM response. Our hypotheses were partially supported. Contrary to initial expectations, protracted amygdalar responses during threat trials of the Protracted Emotion Processing Task did not predict ABM outcome. However, consistent with our hypotheses, results indicated that sustained bilateral amygdala activity during neutral trials predicted poorer outcomes following ABM treatment in anxious patients, as defined by pre- to post-treatment changes in patient-reported symptoms of vigilance. We found that after a neutral word was presented briefly for 300 ms, a subset of anxious individuals appeared to have protracted bilateral amygdalar response to that information, even when they participated in a subsequent non-emotional distractor task. Critically, protracted processing in the bilateral amygdala was correlated with higher residual symptoms of vigilance following ABM treatment, providing evidence that protracted amygdalar response was a significant neural predictor of poorer ABM outcomes. This effect appeared specific to ABM as amygdalar response did not significantly predict improvement in the sham treatment.

While the current findings provide direct insight into *who* might respond poorly to ABM (i.e., those who experienced protracted bilateral amygdalar response in the aftermath of neutral stimuli), prior research can provide context as to *why* amygdalar response to neutral stimuli may be such a salient predictor of ABM outcome. Specifically, although fMRI research in anxious populations has consistently shown dysfunction in circuits involving the amygdala in response to threat^24^, there is also evidence to suggest that some anxious individuals exhibit similar disruptions in these circuits while processing neutral information^30,31^. The amygdala is an important contributor to potential biases for neutral stimuli among anxious individuals because it is responsible for interpreting and encoding the affective properties of stimuli^25^. Because ABM focuses on training attention away from threatening stimuli and toward neutral stimuli, it may not be well suited for anxious individuals who exhibit protracted amygdalar response after only a benign stimulus has been presented, as these individuals may react to neutral information in their environment as a salient and/or worrisome threat due to an overly threat-oriented appraisal of the environment.

The current study’s Protracted Emotion Processing Task may have been particularly well-suited to assess individual differences in salience detection, as the initial prompt of “Does it worry you?” presented briefly at the start of each trial may have primed those who have difficulty distinguishing between neutral and threatening stimuli to exhibit higher amygdalar responses throughout the neutral trials. Alternatively, the task design may have led some patients to experience increased amygdalar reactivity specifically in response to the word “worry”, during both threat and neutral trials. However, in either case, only amygdalar response during neutral trials predicted ABM outcome, suggesting that individuals who are unable to suppress protracted amygdalar responses in the aftermath of objectively neutral stimuli may exhibit saliency biases that are not well-addressed by ABM. Further, this pattern was only apparent in amygdalar reactivity, and not in subjective responses to the neutral stimuli, suggesting that neural predictors of ABM treatment response may be more sensitive than self-report predictors, perhaps due to the reporting biases inherent to self-report indices. However, it should be noted that the subjective worry ratings made during the task were made using a restricted range and thus likely not especially sensitive to fine-grained subjective differences. Future research would benefit from a more sensitive self-report of worry during the task.

Notably, the current results further extend research suggesting that neural responses to neutral stimuli play an integral role in the development and maintenance of anxiety disorders^30,31^. Although the vast majority of ABM research has focused on transient responses to negative stimuli, the current findings suggest that protracted neural responses to neutral stimuli may limit the effectiveness of ABM as they violate the implicit contingencies of the treatment. Specifically, ABM treatments assume that patients will be able to quickly and accurately discriminate between neutral and threat stimuli given that ABM seeks to reallocate attention toward neutral rather than threatening stimuli in early stages of attention. Thus, individuals who have difficulty discriminating between neutral versus threatening stimuli, at the level of amygdalar reactions, may be less able to learn ABM contingencies, which could limit the effectiveness of the intervention.

Taken together, the results from the current study and prior work suggest that there are at least two critical mechanistic predictors of ABM. First, because ABM seeks to target initial attentional allocation to threat (i.e., attentional vigilance), patients who exhibit greater early neural and behavioral responses to threatening stimuli display superior outcomes following ABM treatment because their symptoms are well-suited to the mechanistic intervention target^13,15,16^. Second, as seen in the current study, because ABM retrains attention to be systematically directed towards neutral stimuli, individuals who exhibit protracted levels of amygdala activation in the aftermath of benign stimuli are likely to be poor mechanistic candidates for this intervention as ABM does not address processing biases related to neutral stimuli. Of note, findings from Price et al.^15^. and the current study were conducted using the same sample and thus provide opportunities for direct comparisons of these mechanistic candidates. Whereas the task used in Price et al.^15^. measured transient and sustained responses that occur during the continuous presentation of threatening and neutral images, the Protracted Emotion Processing Task from the current study measured processing that occurs after a threatening or neutral word has been removed and that persists even during a distracting cognitive task. While larger transient amygdalar responses to threatening images predicted superior ABM outcomes in the Price et al.^15^. study, protracted amygdalar responses in the aftermath of neutral words predicted worse ABM outcomes in the current study. These two discrete features of neural processing were not significantly correlated within the present sample. Together, these findings suggest that transient responses to threat stimuli and protracted processing in the aftermath of neutral stimuli are two critical and distinct individual characteristics of neural processing that each impact ABM effectiveness.

It is important to note that the current findings were specific to pre- to post-treatment changes in patients’ self-reported symptoms of vigilance, and our findings did not extend to changes in pre- to post-treatment clinician-rated symptoms of vigilance. These findings were contrary to hypotheses and notable given that clinician-rated measures have shown more reliable and consistent effects of ABM compared to self-report^14^. In the current study, disparate findings for clinician-rated versus self-report primary outcome measures could be in part due to differences in the specific aspects of vigilance assessed by the two measures. Specifically, the self-report measure of vigilance (i.e., MASQ Anxious Arousal subscale) mainly focused on psychosomatic symptoms that are experienced internally and pervasively in daily life. In contrast, the clinician-rated measure of vigilance (i.e., CAPS) more specifically assessed how the patient monitored threat in the external environment. Because the Protracted Emotion Processing Task focused on internal perseveration about potential threats not yet materialized in the external environment (i.e., “Does it worry you?”), neural predictors from this task may have been better suited to predict more internally-experienced symptoms of vigilance and/or more generalized patterns of somatic anxiety symptoms, which was best assessed by the self-report measure. Future research will benefit from the addition of both self-report and clinician-rated indices that can simultaneously measure and parse symptoms of vigilance experienced during internal versus external states.

There were several limitations of the current study that highlight areas for future research. First, although a strength of the study was the use of random assignment to active ABM versus the sham condition, the uneven allocation protocol that favored ABM assignment reduced power to examine neural predictors of treatment response in the sham condition. Relatedly, sham ABM (as defined in the current study) could be conceptualized as merely a lower dose of active ABM (i.e., 50/50 threat versus neutral probe contingency) and thus similar mechanisms may impact outcomes during lower-dose ABM, albeit with weaker effects. Thus, while there is preliminary evidence that the neural predictors of treatment response were specific to the active, full-dose ABM group, future research would benefit from larger samples to further test placebo and dosing effects or spontaneous recovery. Second, we selected and analyzed only two brain regions a priori (i.e., left and right amygdala) and therefore may be overlooking other parts of the brain where there could be significant correlations. While this approach preserves power by limiting the number of multiple comparisons (particularly given the need in these timeseries analyses to correct for multiple comparisons over time), future, larger studies could employ whole brain analyses to identify dysregulated anxiety circuits that predict ABM outcomes. Finally, while the current study was designed to examine neural predictors of ABM outcomes in a controlled laboratory setting, future work should focus on the translation and dissemination of these findings into the clinical setting, including identification of predictors that do not rely on costly fMRI assessments.

In conclusion, the current study provides preliminary evidence that protracted hyperactivity in the amygdala can be used to predict which anxious patients are most likely to benefit from ABM. Specifically, we found that protracted activity in the amygdala in the aftermath of a briefly presented neutral word, which persisted during a subsequent non-emotional distractor, may interfere with successful outcome in ABM. The results of the current study have both important clinical and research implications. For example, these findings add to a growing body of research that suggest there are critical individual difference factors that impact the efficacy of ABM (see also 13–16) and that identifying individual differences that predict ABM response may be the key to understanding the inconsistency of efficacy findings in the ABM literature more broadly^14^. From a precision medicine standpoint, this type of individual mechanistic assessment may allow for identification of specific subsets of anxious patients who would be good candidates for ABM. This would be particularly useful if fMRI predictors can be translated into a clinically available form^39^. In addition, existing cognitive intervention approaches could be supplemented or refined to target protracted forms of neutral stimulus processing, which have not been a focus of prior attention retraining efforts, but may represent an opportunity to reduce overgeneralized threat perception biases in anxiety. Together, these strategies could potentially increase response rates and thus improve care for individuals with anxiety disorders.

## Supplementary information

Supplement

## Data Availability

The Pupil Toolkit and in-house fMRI MATLAB code was used to implement the analytic plan. Details regarding this code and its availability can be found at www.pitt.edu/~gsiegle/.
