# Supplementary material for: Protracted amygdalar response predicts efficacy of a computer-based intervention targeting attentional patterns in transdiagnostic clinical anxiety
Source: Transl Psychiatry. 2019 Mar 28;9:121. doi: 10.1038/s41398-019-0458-x (PMC6438974; doi:10.1038/s41398-019-0458-x)
Supplement: Supplementary file 1 — Supplement [file 41398_2019_458_MOESM1_ESM.docx]

**Protracted Amygdalar Response Predicts Efficacy of a Computer-Based Intervention Targeting Attentional Patterns in Transdiagnostic Clinical Anxiety**

***Supplemental information***

Supplemental Materials and Methods 2

Inclusion/Exclusion Criteria 2

CONSORT Diagram 3

Secondary Clinical Outcomes 4

Selection of Idiographic Words 4

Behavioral Ratings During the Protracted Emotion Processing Task 4

Table S1. Split-half Reliability for Amygdalar Responses 6

Supplemental Analyses 7

Analyses of Secondary Clinical Outcomes 7

Effectiveness of ABM Across the Sample 7

Analyses of the Relation between Behavioral Ratings and Other Study Variables 7

Figure S1. Amygdalar Responses among Patients in the Sham Condition 9

Supplemental References 11

# Supplemental Materials and Methods

## Inclusion/Exclusion Criteria

All patients displayed clinical levels of transdiagnostic anxiety at the baseline assessment as defined by two inclusion criteria. Specifically, patients had to endorse often feeling anxious, shy or worried, as defined by scores > 45 on the Spielberger State-Trait Anxiety Inventory trait form (STAI-T) (1) and exhibit clinically significant impairment as indicated by scores at or above 75^th^ percentile on the WHO Disability Assessment Schedule 2.0 (WHODAS 2.0) (2). These two criteria were designed to get an appropriate distribution of individuals with clinically impairing transdiagnostic anxiety. Patients were included in the study if they (a) passed the first two criteria; (b) were not currently participating in cognitive behavioral therapy (CBT); (c) were not taking any psychotropic medications; (d) met standard fMRI inclusion criteria; (e) had no evidence of bipolar, psychotic, autism spectrum, substance dependence, or primary depressive disorder; (f) showed no evidence of acute suicidality; (g) scored >20/40 on the Snellen test indicating normal or corrected-to-normal vision; and (h) had a reading level >6^th^ grade as per the Wide Range Achievement Test – Revised (WRAT-R) reading scale (3).

## CONSORT Diagram

Missing data

- fMRI data (n = 5)
- Onset of psychosis (n=1)

Analysed (n= 39; 87%)

Missing data

- fMRI data (n=2)

Analysed (n= 19; 90%)

## Analysis

Discontinued intervention (n= 4)

- Withdrew voluntarily (n= 4)

Completed intervention and post-assessment (n=45; 92%)

Discontinued intervention (n= 0)

Completed intervention and post-assessment (n=21; 100%)

## Follow-Up

## Allocation

Assessed for eligibility (n= 100)

Randomized (n= 70)

Excluded (n= 30)

♦  Not meeting inclusion criteria (n= 21)

♦  Declined to participate (n= 9)

Allocated to ABM intervention (n= 49)

♦ Received allocated intervention (n= 49)

Allocated to control intervention (n= 21)

♦ Received allocated intervention (n= 21)

## Enrollment

## Secondary Clinical Outcomes

Due to the majority of patients meeting diagnostic criteria for Generalized Anxiety Disorder at baseline (83% of the sample, according to Mini International Neuropsychiatric Interview; MINI), the Penn State Worry Questionnaire (PSWQ) (5), was used to assess the severity and controllability of GAD’s defining symptom (worry) as a secondary outcome. In addition, the General Distress subscale of the MASQ was used to assess levels of general distress that are non-specific to anxiety disorders as an additional secondary outcome. In the current study, both the PSWQ and the MASQ General Distress Subscale exhibited excellent internal consistency (αs ranging from .91-.92).

## Selection of Idiographic Words

At baseline, ten idiographic threat words were selected collaboratively by the participant and clinical interviewer, immediately following the baseline clinical interview. These words were idiographically chosen in order to capture the full range of concepts most relevant to the participant’s daily experience of anxiety. All idiographic items were rated by the participant as -2 or -3 on a scale of pleasantness ranging from +3 (“very pleasant”) to -3 (“very unpleasant”). Each idiographic threat word was paired, on an individual (per-participant) basis, with a neutral word (drawn from a normative corpus) that was rated as neutral (score=0) by the participant and matched to the idiographic threat word on word length and the participant’s familiarity rating (on a 7-point Likert-like scale).

## Behavioral Ratings During the Protracted Emotion Processing Task

During the Protracted Emotion Processing Task, patients were instructed to rate their level of worry about the presented word using buttons assigned for “Yes,” “Somewhat,” and “No.” The average percentage of trials that the patients responded “yes”, “somewhat”, and “no” or gave a non-response for neutral and threat trials is presented below.

|  | “Yes” | “Somewhat” | “No” | Non-Response |
| --- | --- | --- | --- | --- |
| Neutral | 4% | 15% | 80% | 1% |
| Threat | 79% | 17% | 3% | 1% |

## Table S1. Split-half Reliability for Amygdalar Responses

# Supplemental Analyses

## Analyses of Secondary Clinical Outcomes

Neither left nor right amygdala activity during neutral or negative trials was significantly associated with reduced general distress (MASQ General Distress scores) or worry (PSWQ scores) following ABM or sham (consecutive *r*s < .32).

## Effectiveness of ABM Across the Sample

As originally published in a prior manuscript from the same sample (4), among participants in the ABM condition, CAPs scores significantly decreased from pre- [*M* = 4.54 (*SD* = 2.03)] to post-intervention [*M* = 3.90 (*SD* = 1.97)] (*p* = .017; *d* = .32). Similarly, MASQ anxious arousal scores significantly decreased from pre- [*M* = 32.20 (*SD* = 10.66)] to post-intervention [*M* = 27.41 (*SD* = 8.59)] (*p* < .001; *d* = .47) in the ABM condition. Among participants in the sham condition, CAPs scores significantly decreased from pre- [*M* = 5.24 (*SD* = 2.23)] to post-intervention [*M* = 4.19 (*SD* = 2.16)] (*p* = .011; *d* = .48). In contrast, MASQ anxious arousal scores did not significantly decrease from pre- [*M* = 34.05 (*SD* = 10.72)] to post-intervention [*M* = 30.29 (*SD* = 13.92)] (*p* = .116; *d* = .29) in the sham condition.

## Analyses of the Relation between Behavioral Ratings and Other Study Variables

To examine the relation between behavioral worry ratings and 1) amygdalar responding and 2) primary outcomes, proportion scores were created to address the non-normal distribution of subjective behavioral worry ratings collected during the Protracted Emotion Processing Task. Specifically, for neutral trials, proportion scores were calculated to determine what proportion of the trials the patient answered “no” to the “Does it worry you?” prompt. For threat trials, proportion scores were calculated to determine what proportion of the trials the patient answered “yes” to the prompt. Nonresponses were not included in the proportion counts.

For the MASQ Anxious Arousal subscale, residual change was not significantly related to proportion scores during neutral (*r* = -.15, *p* = .26) or negative (*r* = -.01, *p* = .92) trials. For the CAPS, residual change was not significantly related to proportion scores during neutral (*r* = -.23, *p* = .10) or negative (*r* = -.06, *p* = .68) trials. Finally, as reported in the main text, neither left nor right amygdala activity during neutral or threat trials was significantly associated with proportion scores during neutral or threat trials, respectively (consecutive *r*s < .32)**.**

## Figure S1. Amygdalar Responses among Patients in the Sham Condition

a

**
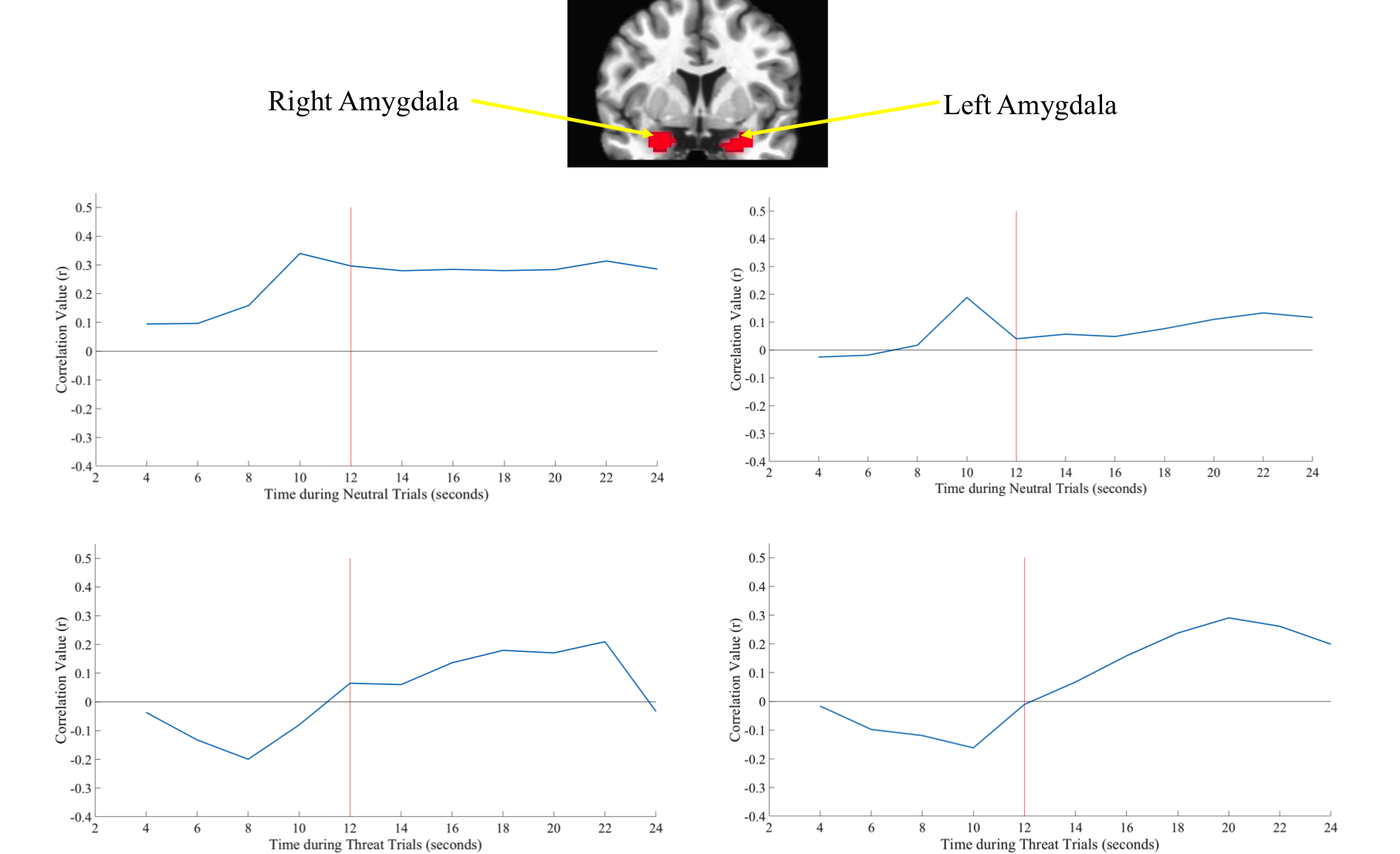
**

b

c

*Figure S1 Legend.* (a). Coronal view of the right and left amygdala ROIs (b). Correlation coefficient (r) value between MASQ Anxious Arousal residual scores and BOLD activity in the left and right amygdala across the full time course of neutral word trials. The vertical red line at 12s separates the emotion processing portion of the Protracted Emotion Processing Task from the digit memory portion. (c). Correlation coefficient (r) value between MASQ Anxious Arousal residual scores and BOLD activity in the left and right amygdala across the full time course of threat word trials.

*Note.* Time points at which correlation coefficients were considered significant would be highlighted in red on the x axis. However, even when using lenient significance test thresholding (as described in the main text), there were no significant correlations between left or right amygdala activity and anxious arousal MASQ residual scores across sham patients during the neutral or threat trials.

# Supplemental References

1. Spielberger CD. State-Trait Anxiety Inventory. John Wiley & Sons, Inc.; 2010.

2. Ustun TB. Measuring health and disability: Manual for WHO disability assessment schedule WHODAS 2.0. 2010.

3. Jastak SR, Wilkinson GS. WRAT-R: Wide range achievement test-revised administration manual. Jastak Associates; 1984.

4. Price RB, Cummings LR, Gilchrist D, Graur S, Banihashemi L, Kuo S, et al. Toward personalized, brain-based behavioral intervention for transdiagnostic anxiety: Transient neural responses to negative images predict outcomes following a targeted computer-based intervention. J Clin Consult Psychol. 2018.

5. Meyer TJ, Miller ML, Metzger RL, Borkovec TD. Development and validation of the Penn State Worry Questionnaire. Behav Res Ther. 1990;28:487–95.
